# Facial Emotion Analysis During Rehabilitation Treatment of a Patient With Acute Generalized Peritonitis and Delirium

**DOI:** 10.7759/cureus.94004

**Published:** 2025-10-07

**Authors:** Naoto Seriu, Shogo Sasaki, Yuya Mawarikado, Yusuke Inagaki, Akira Kido

**Affiliations:** 1 Rehabilitation Medicine, Nara Medical University, Kashihara, JPN; 2 Medicinal Biology of Thrombosis and Hemostasis, Nara Medical University, Kashihara, JPN

**Keywords:** artificial intelligence (ai), emotion analysis, facial expression analysis system, post operative delirium, postoperative physiotherapy rehabilitation

## Abstract

Rehabilitation treatment is known to improve not only physical function but also emotional well-being. However, objectively assessing emotions in critically ill patients with impaired consciousness by using conventional subjective measures is difficult. In this study, we focused on a specific method for analyzing emotions from facial expressions and aimed to perform facial emotion analysis in a single patient with delirium. The patient was a man in his 70s with acute generalized peritonitis who developed postoperative delirium. Rehabilitation treatment was initiated on the first day after surgery, and facial emotion analysis was performed before and after rehabilitation treatment on day 14. Five emotional factors (Neutral, Happy, Sad, Angry, and Surprised) were measured, and 38 measurements were taken during 19 sessions. Visual analog scale (VAS) and salivary α-amylase activity (sAA) levels were also measured 22 times in 11 sessions from day 30 until discharge. Throughout the imaging period, the Happy emotion increased significantly after training when compared with the period before training, whereas the Surprised emotion decreased significantly. Furthermore, during the delirium period, happiness showed an increasing trend following training, whereas other emotional factors tended to decline. The VAS score decreased after training, whereas sAA levels did not show a consistent change. These observations suggest that facial emotion analysis may be feasible as an objective adjunct, but its clinical utility and generalizability require confirmation in larger studies.

## Introduction

In acute care settings, early rehabilitation improves both physical function and psychological outcomes in critically ill ventilator-dependent patients [1]. Early rehabilitation has been beneficial in various clinical settings such as joint replacement surgery [2,3], thoracoabdominal surgery for esophageal and gastric cancer [4], and catheter aortic valve implantation [5]. Studies reported improvements in both physical function and psychological status, indicating the role of rehabilitation in supporting multiple aspects of patient recovery. These studies primarily relied on self-reported assessments of perceived health status and well-being. However, such self-reported measures are difficult to obtain in critically ill patients who develop altered consciousness or delirium. Delirium is characterized by an acute, fluctuating disturbance of attention and cognition and is typically reversible; however, it can impede the delivery of acute care and participation in early rehabilitation. In such a situation, self-reporting is often unreliable or unobtainable [6-8]. Quantitative evaluation of emotional and psychological expressions in such patients may aid in adjusting rehabilitation intensity daily and further support the clinical efficacy of early rehabilitation. Therefore, an objective method of assessing emotional expressions is required.

Recently, methods were developed to analyze emotions based on human facial expressions and voices. As an example of a medical application, facial emotion analysis during cardiopulmonary exercise testing in patients with coronary artery disease showed that testing increased negative emotions and decreased positive ones [9]. Another group reported that analysis of facial expressions during a speech task in 10 patients with traumatic brain injury (TBI) revealed a significantly higher percentage of engagement time (defined by multiple facial action units) and smile time in patients with traumatic injury when compared with control participants [10]. Patients with TBI may compensate for communication impairments, such as reduced language output and memory function, by using facial expressions more frequently. Therefore, we hypothesized that facial emotion analysis, as a noninvasive, quantitative, and objective assessment of emotion, may be useful in assisting rehabilitation treatment.

This study aimed to investigate whether emotions can be quantified by capturing the facial expressions of critically ill patients undergoing rehabilitation treatment using emotion analysis software. We analyzed the case of a patient who was admitted to the intensive care unit (ICU) after undergoing surgical treatment during his hospital stay and developed postoperative delirium.

## Case presentation

Initial presentation and clinical course

The patient was a man in his 70s who was previously independent in all activities of daily living (ADL). He presented with sudden-onset abdominal pain of several hours’ duration and underwent emergency surgery the same day. A diagnosis of acute generalized peritonitis due to sigmoid colon perforation was made, and emergency open sigmoid colon resection, creation of a descending colostomy, and intraperitoneal lavage and drainage were performed. He developed septic shock and disseminated intravascular coagulation, required mechanical ventilation, and was admitted to the ICU for continuous hemodiafiltration.

On rehabilitation day 1, the Glasgow Coma Scale (GCS) score was E3VTM6 [11]. The patient remained ventilated, and the Richmond Agitation-Sedation Scale (RASS) was -3 (moderately sedated) [12]. Ward nurses conducted delirium assessments during both day and night shifts using the Confusion Assessment Method for the ICU (CAM-ICU) when the patient was in the ICU and the Confusion Assessment Method (CAM) in the general ward [6,7]. The CAM-ICU assessment results were positive on day 2. The patient was extubated on day three and trained to sit, stand, and transfer to a wheelchair. The patient was discharged from the ICU on day 11. On that day, examination revealed GCS E4V4M6, a nasal cannula at 1.5 L/min, manual muscle testing grade 5 in both upper limbs and grade 4 in both lower limbs, and a Functional Independence Measure (FIM) score of 27/126 [13,14]. The CAM ratings were positive from days 2 to 21, after which they were negative. During the delirium period, the RASS fluctuated between -4 and +2, and bedside observation revealed difficulty in sustaining attention with a fluctuating course. During the non-delirium period, the RASS was stable at 0.

The patient was selected for this study for three reasons: (1) he was experiencing delirium, (2) he was able to follow the directions of the physiotherapist, and (3) he met the requirements for facial expression analysis without facial injury. This study was approved by the Ethics Review Committee of our university (approval number: 3751). Written informed consent was obtained from the patient and his wife. This study was conducted in accordance with the ethical standards of the Declaration of Helsinki and the Japanese law.

Delirium and non-delirium periods

Emotions can be evaluated using subjective scales; however, such methods cannot be used when consciousness is impaired, including in delirium. Therefore, this study aimed to establish an objective method. Because the processing of facial expression is likely to differ between the delirium and non-delirium periods, we compared facial emotion analyses between these two states. Delirium was determined from medical records, and patients were classified as delirious when either the CAM-ICU or the CAM was positive.

Rehabilitation protocol

Rehabilitation commenced on the first postoperative day of the treatment period and was administered five times per week for 20-40 min per session. The rehabilitation treatment regimen was tailored to the patient's daily condition and comprised muscle-strengthening training, primarily targeting the lower limb muscles, and getting out of bed (sitting, wheelchair transfer, and walking). Throughout the rehabilitation process, training was conducted by removing physical restraints. Once the patient demonstrated improved activity and was able to access the training room, step-climbing and aerobic exercises were performed using a bicycle ergometer.

Measurement protocol

**Figure 1:**
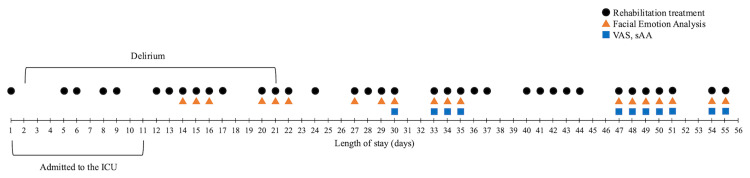
Schedule with rehabilitation treatment, facial emotion analysis, VAS, and sAA. ICU: intensive care unit; VAS: visual analog scale; sAA: salivary α-amylase activity

The measurement schedule is illustrated in Figure 1. During hospital stay, 36 rehabilitation sessions were administered (black dots). Facial emotion analysis was performed from day 14 and conducted 38 times at the start and end of the 19 rehabilitation sessions (red triangles). For subjective stress assessment, a visual analog scale (VAS) was used from day 30. For objective stress assessment, salivary α‑amylase activity (sAA) levels were measured using a salivary amylase monitor (Nipro Co., Osaka, Japan) from day 30 (blue squares). Facial emotion analysis was conducted using the MAL Face Emotion software (Vitalify Asia Co., Ltd., Ho Chi Minh City, Vietnam), which uses an artificial intelligence model to detect facial expressions in real time from video recordings. The software assigned scores of 0-100 for Neutral, Happy, Sad, Angry, and Surprised emotions, which were normalized to percentages totaling 100%. The model, which is based on MobileNets and trained primarily on AffectNet, detects expressions at 15-20 frames per second [15,16]. Videos were recorded on an iPad (Apple, Cupertino, CA, USA) and analyzed during 30-s interviews before and after the rehabilitation sessions. The patient was asked routine questions about their condition, sleep, and fatigue, which were not expected to influence their emotions.

Because the data were non-normal (Shapiro-Wilk test), we used Wilcoxon signed-rank tests for all variables (facial emotion scores, VAS, and sAA) to compare the pre-and post-training values. We hypothesized that the process of facial expression may differ according to delirium status. Therefore, we stratified analyses into delirium and non-delirium groups. For facial emotion analysis, the 19 sessions were further grouped into delirium (n = 5; onset of delirium) and non-delirium (n = 14; no delirium) periods. Statistical analysis was performed using JMP Student Edition 18.2.0 software (SAS Institute Inc., Cary, NC, USA), and P-values less than 0.05 were considered statistically significant.

Clinical outcomes

**Figure 2:**
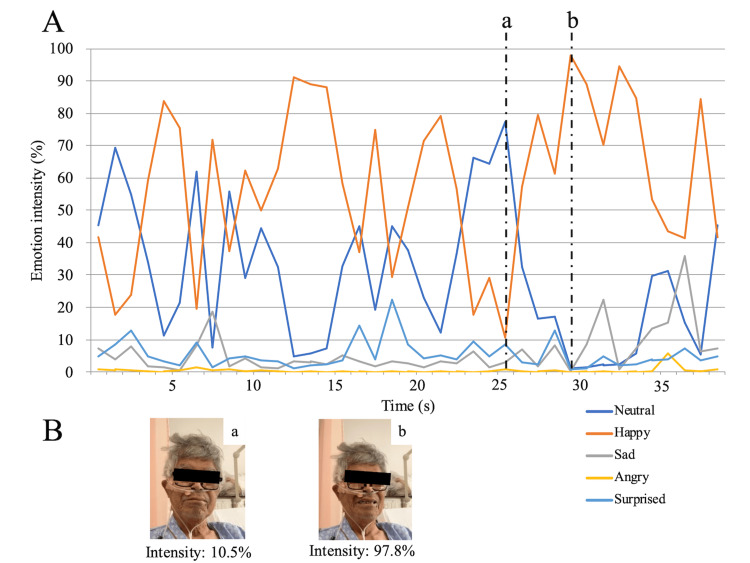
A shows the change of each emotional factor in real time. B shows the face images at low (a) and high (b) intensities of the Happy emotion.

After qualitatively confirming that the patient’s facial expressions were changing, we began recording on day 14 and conducted a facial emotion analysis based on the data. Figure 2A shows representative data from the facial emotion, with a graph showing real-time changes in emotions. Figure 2B presents representative facial images corresponding to (a) low and (b) high happiness intensities of the Happy emotion. The time required to acquire the images was 25.57 ± 16.56 s before and 29.26 ± 10.48 s after training (mean ± SD).

**Figure 3:**
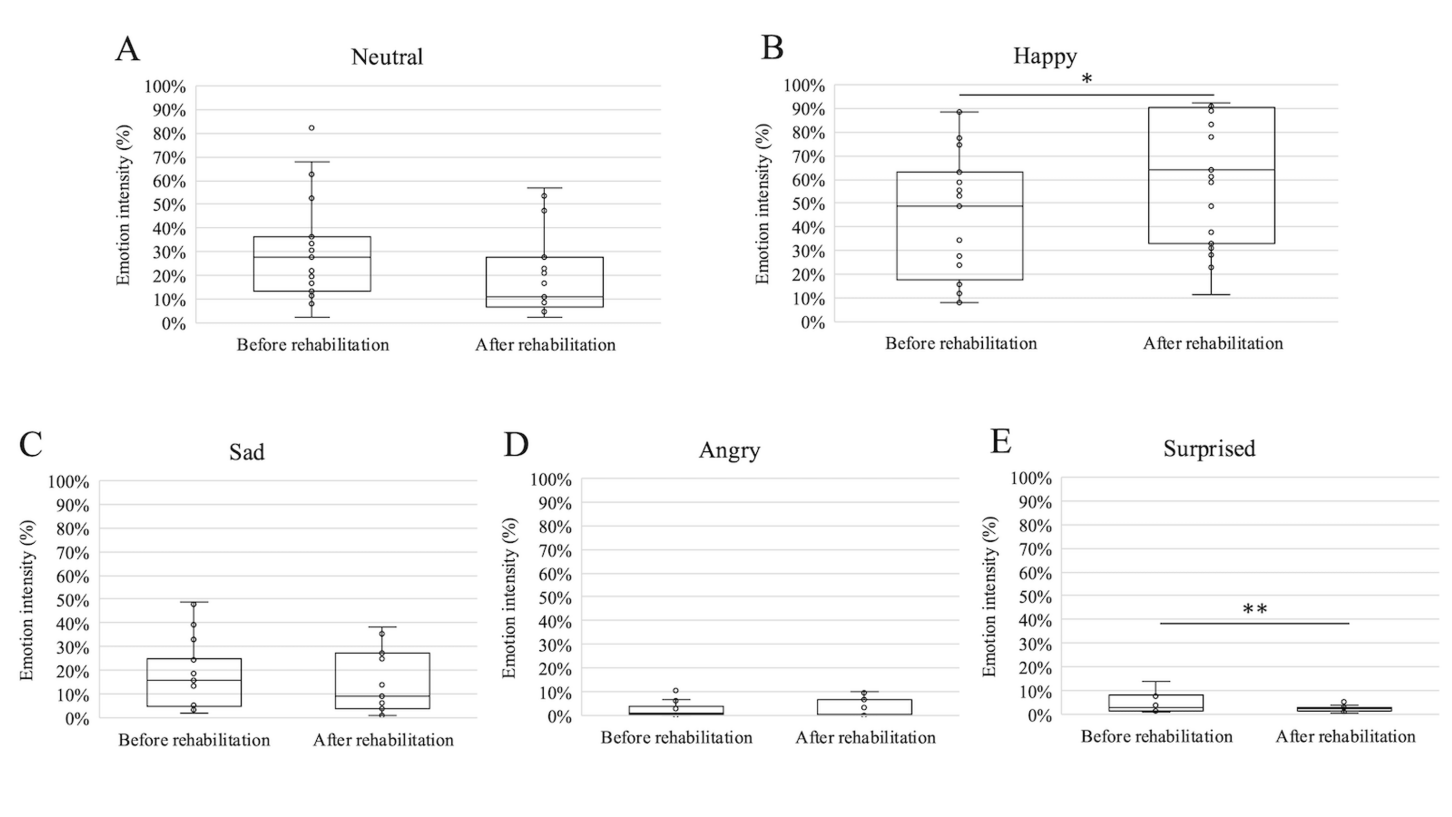
: The box plots from A to E show the scores for the five emotions (Neutral, Happy, Sad, Angry, and Surprised) for all sessions before and after the rehabilitation treatment, with the horizontal line representing the median and the circles representing the scores for each session. *p = 0.049; **p = 0.008

**Figure 4:**
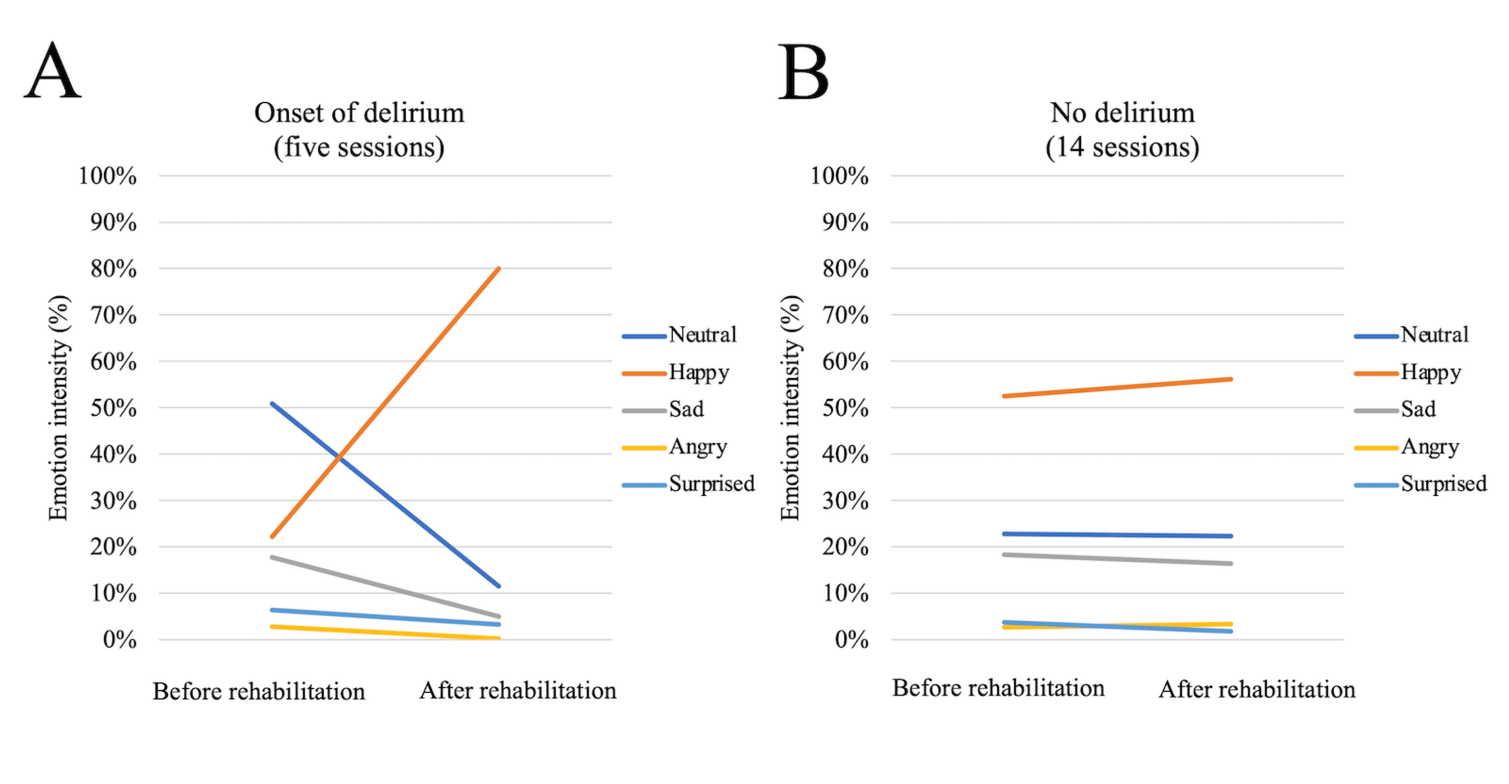
A is the median value of each emotional factor before and after rehabilitation treatment during the five sessions in which delirium developed throughout the measurement period, and B is the median change in each emotional factor before and after rehabilitation treatment during the period without delirium.

Box plots are shown for each emotional factor for all recording periods (19 sessions, 38 times) of the facial emotion analysis (Figure 3A-3E). The Happy emotion (Figure 3B) increased by 17.92 percentage points (95% confidence interval (CI), −0.05 to 35.88; P=0.049), whereas the Surprised emotion (Figure 3E) decreased by 2.26 percentage points (95% CI, −3.93 to −0.58; P=0.008). Notably, when focusing on the five sessions in the delirium-onset period, all emotion factors except the Happy emotion tended to decrease after training, while it alone showed a clear upward trend (Figure 4A). In the sessions without delirium, no consistent changes were observed in any of the affected factors (Figure 4B).

**Figure 5:**
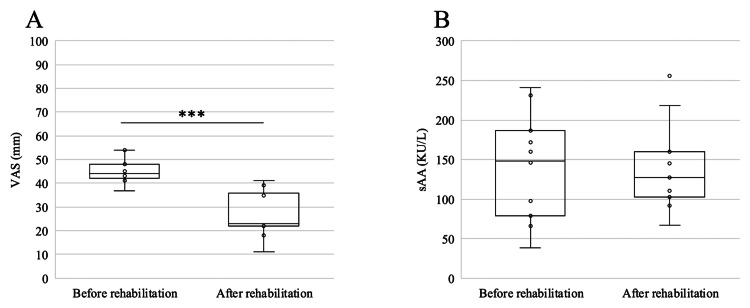
Box plots show changes in VAS (A) and sAA (B) levels before and after rehabilitation treatment. VAS: visual analog scale; sAA: salivary α-amylase activity ***p = 0.001

During the delirium-free period (Figures 5A-5B), the VAS score decreased by 18.6 mm (95% CI, -23.2 to -14.1; P=0.001), whereas sAA decreased by 3.18 KU/L (95% CI, -65.8 to 59.4; P=0.846) and was not significant.

The patient recovered well with an improved FIM score of 107/126 points. Manual muscle testing showed that his muscle strength improved to grade 5 (normal) in all four limbs, and he was discharged from the hospital on day 56 after being able to walk independently.

## Discussion

We attempted to apply human facial emotion analysis to rehabilitation medicine. Facial expressions of patients with acute generalized peritonitis who developed delirium during hospitalization were recorded using a tablet device before and after rehabilitation treatment. No adverse events were observed due to the video recording, and the analysis could be performed safely during the delirium period. Specialized software was used to analyze and quantify each emotional factor (Neutral, Happy, Sad, Angry, Surprised). Throughout the imaging period, the Happy emotion increased significantly after training, whereas the Surprise emotion decreased significantly.

Interestingly, until the fifth session at delirium onset (Figure 4A), the Happy emotion showed a trend toward an increase after training, whereas the other factors tended to decrease. We found no significant differences in the individual emotional factors after the disappearance of delirium. Obayashi reported that facial emotion analysis of a patient with a prolonged disorder of consciousness due to severe head trauma revealed that the Happy emotions were expressed by auditory stimuli under various conditions, with values varying for each interesting stimulus [17]. Delirium is an acute-onset disorder of consciousness, characterized by fluctuations in cognitive and attentional functions [6-8]. The Happy emotion was also detected in the present case, which may have been stimulated by the rehabilitation treatment (comfort) during the onset of delirium, suggesting a relationship with the aforementioned report [17]. These results are consistent with those of previous studies. Early mobilization, including seated training and wheelchair transfers, has been shown to shorten the duration of delirium [18]. Benefits from exercise-based interventions have also been reported to be greater among patients who enter hospitalization with higher levels of functional independence [19]. In addition, tailored, multi-component exercise programs have been linked to improved functional recovery and the preservation of cognitive abilities [20]. Viewed together, these lines of evidence suggest that the increase in positive emotional expression seen in this case may represent one facet of the therapeutic value of non-pharmacological approaches to delirium care.

In addition, the VAS and sAA levels were evaluated in 11 of the 14 sessions without delirium (14 sessions). The sAA levels also showed a decreasing trend after training, but the difference was not significant. Thus, facial emotion analysis may be considered an independent method of assessment, distinct from traditional self-reported subjective assessment using VAS and noninvasive objective assessment using sAA, which are often not practical in patients with delirium, as in this case.

Quantifying patient emotions can support the selection of training tasks and the rationale for setting training intensity. Protocols that are developed based on quantified emotions may be better suited to disease status, physical characteristics, and the preferences of patients. In addition, the ability of facial emotion analysis to capture changes in emotion in real time and over time may be an advantage over traditional assessment tools such as VAS and sAA.

The first limitation of our study was the technical difficulty in capturing images during rehabilitation treatment (while performing training tasks). Therefore, it was not possible to determine which specific rehabilitation tasks influenced emotional expressions. Rehabilitation in Japan typically involves face-to-face training. Additionally, in this study, a single physiotherapist performed the recording and training sessions. Rehabilitation treatment in the acute phase is primarily based on early mobilization. Therefore, the development of safer recording methods is necessary. This was a single-patient study, which limited the statistical analysis of the data. Patients admitted to the ICU present multiple disabilities, including complex medical conditions, comorbidities, and treatment-related adverse events, which present a challenge in generalizing the course and progress of rehabilitation treatment from admission to discharge.

## Conclusions

Facial emotion analysis was used to rehabilitate a patient with acute disseminated peritonitis who presented with delirium. This is the first report (to our knowledge) on the use of facial emotion analysis as a novel clinical assessment tool during rehabilitation therapy in patients with delirium. This method was clinically feasible with no adverse events. Facial emotion analysis remains a developing field, grounded primarily in theoretical work, with diverse methodological approaches spanning from image parameterization to data interpretation. This case report suggests that emotion assessment, traditionally reliant on clinician intuition, may provide an objective, quantitative adjunct through facial emotion analysis. Its clinical utility and applicability require confirmation in larger studies.
